# The Hong Kong Infection Control Nurses’ Association – the key to success

**DOI:** 10.1186/2047-2994-4-S1-P59

**Published:** 2015-06-16

**Authors:** RFY Chan, Conita HS Lam, Queenie WL Chan, Chi Ping Chen, Christina WY Cheung, Radley HC Ching, Gloria CS Chiu, Chun Hoi Kan, Shirley SY Lee, Annie FY Leung, Isadora LC Wong, Ida KS Yip, Patricia TY Ching

**Affiliations:** 1 Infection Control Team, HK Hospital Authority Hospitals, Hong Kong, China; 2 HK Sanatorium & Hospital, Hong Kong; 3 Hong Kong Baptist, Hong Kong

## Introduction

The HKICNA was founded in 1989 with only 44 members including advisors. They all shared experiences and a vision with strong will, contributing their own time to promote infection prevention and control. The followings were efforts witnessing the development, impacts to the profession, staff and community. A new norm of infection control was born, working in partners with and contributing to the healthcare system in Hong Kong.

## Objectives

To promote infection control across the boundaries.

## Methods

The objective was achieved through the continuous education by a dedicated group of *assertive* and *committed* nurses who possess *knowledge* and *innovative* ideas *networking* together towards a *humanized* infection control for the health of all by all means.

Period: in 25 years' time since 1989.

## Results

The HKICNA members have expanded to twenty-fold of the original and up to 1400 in 2003-2004. To build up knowledge, training course was organized annually since 1999 which was well recognized by hospitals in Hong Kong with attendances >5000; ad hoc seminars on novel emerging diseases were as needed. In 2002, biannual HKICNA newsletter was first published; to strengthen the network with international authorities, biennial International Conference was organized in collaboration with local nursing associations from 2004 onwards; which served as a platform for experience sharing from 800 – 1000 overseas’ delegates in each event. The webpage was developed in 2007 to enhance communication and promotion. Participation in health carnival in 2010 broadened the scope to community involving public. There were innovative activities e.g. research grant award, sponsorship and scholarship, hand hygiene poster design competition and video on “Hand Hygiene Dance” for different generations to enhance sustainability.

## Conclusion

With the support from our healthcare workers and public, and guidance from renowned advisors; HKICNA, with all the education and promotion works, has been growing more influential in the healthcare system in Hong Kong and also serves as a bridge for the overseas. The association stands for Humanity, Knowledge, Innovation, Commitment, Networking and Assertiveness; which is the key to success.

## Disclosure of interest

None declared.

